# Anti-Ri-associated paraneoplastic ophthalmoplegia-ataxia syndrome in a woman with breast cancer: a case report and review of the literature

**DOI:** 10.1186/s13256-020-02410-z

**Published:** 2020-06-12

**Authors:** Giuseppe Sena, Gaetano Gallo, Giuseppina Vescio, Denise Gambardella, Stefano de Franciscis, Mariuccia Renne

**Affiliations:** Department of Medical and Surgical Sciences, U.O. of General Surgery, University of Catanzaro, Viale Europa, 88100 Catanzaro, Italy

**Keywords:** Breast cancer, Paraneoplastic neurological syndromes, Ophthalmoplegia-ataxia syndrome, Cerebellar paraneoplastic degeneration

## Abstract

**Background:**

Breast cancer is the most common cancer in women. However, in the management of breast cancer, paraneoplastic neurological syndromes represent a diagnostic and therapeutic challenge. The diagnosis of paraneoplastic neurological syndromes is difficult due to the heterogeneity of symptoms, the timing of presentation, and the absence of antibodies, and it generally occurs before the diagnosis of breast cancer in 80% of patients who develop paraneoplastic neurological syndromes. We describe a 72-year-old woman with subacute ophthalmoplegia-ataxia syndrome who was subsequently diagnosed as having breast cancer and anti-Ri antibodies.

**Case presentation:**

A 72-year-old post-menopausal Caucasian woman, with a positive medical history for diabetes mellitus and hypertension, presented with a 3-month onset of blurred vision, diplopia, and progressive gait disturbance. Serological tests were positive for well-characterized onconeural antibodies (anti-Ri). A whole-body computed tomography scan revealed a nodular opacity under her left nipple and axillary adenopathy. A biopsy of her left breast was performed, and histological examination showed ductal carcinoma. She underwent a superoexternal quadrantectomy with left axillary dissection. The final diagnosis showed infiltrating ductal carcinoma of the breast (T1c N1 M0, stage IIA) associated with paraneoplastic ophthalmoplegia-ataxia syndrome. At a 6-month follow-up, she showed no clinical or instrumental evidence of neoplastic recurrence with partial clinical improvement of neurological symptoms, such as ataxia and diplopia.

**Conclusion:**

The diagnosis of paraneoplastic neurological syndromes is often late, as in this patient, but treatment at an early stage may provide a good prognosis. Furthermore, this is one of several cases of an anti-Ri paraneoplastic neurological syndrome not associated with myoclonus, which reinforces the belief that opsoclonus myoclonus syndrome is not pathognomonic of the associated anti-Ri paraneoplastic neurological syndromes.

## Background

Breast cancer is the most common cancer in women comprising 10.4% of all cancers among women worldwide. The mortality rate has decreased by 34% in the last 30 years, demonstrating a significant improvement in diagnosis and treatment [1, 2].

However, in the management of breast cancer, paraneoplastic neurological syndromes (PNSs) represent a diagnostic and therapeutic challenge. PNSs classically occur with cerebellar symptoms such as ataxia, nystagmus, and dysarthria and with peripheral neuropathies, stiff person syndrome, encephalomyelitis (including limbic encephalopathy), and paraneoplastic retinopathy. PNSs are the consequence of the natural immune response against neoplastic antigens. In fact, the ectopic expression of neural antigens induces a response against neurons expressing the shared antigen with the tumor [3]. The diagnosis of PNSs is difficult due to the heterogeneity of symptoms, the timing of presentation, and the absence of antibodies, and it generally occurs before the diagnosis of breast cancer in 80% of patients who develop PNSs [4]. Breast cancers associated with PNSs are more aggressive and have a poor prognosis [5], but few reports in the literature have reported a correlation between anti-Yo antibodies and human epidermal growth factor receptor 2 (HER2)-positive breast cancer [6]. We describe a 72-year-old woman with subacute ophthalmoplegia-ataxia syndrome who was subsequently diagnosed as having breast cancer and anti-Ri antibodies.

## Case presentation

**Fig. 1:**
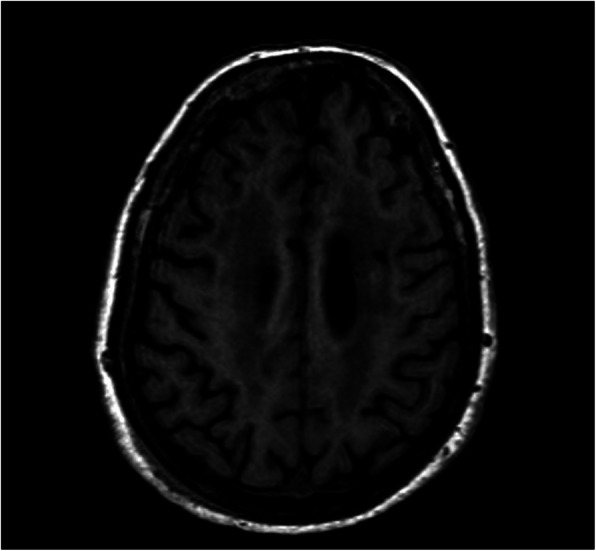
Magnetic resonance imaging revealed a diffuse periventricular (deep and cerebellar) white matter alteration

**Fig. 2:**
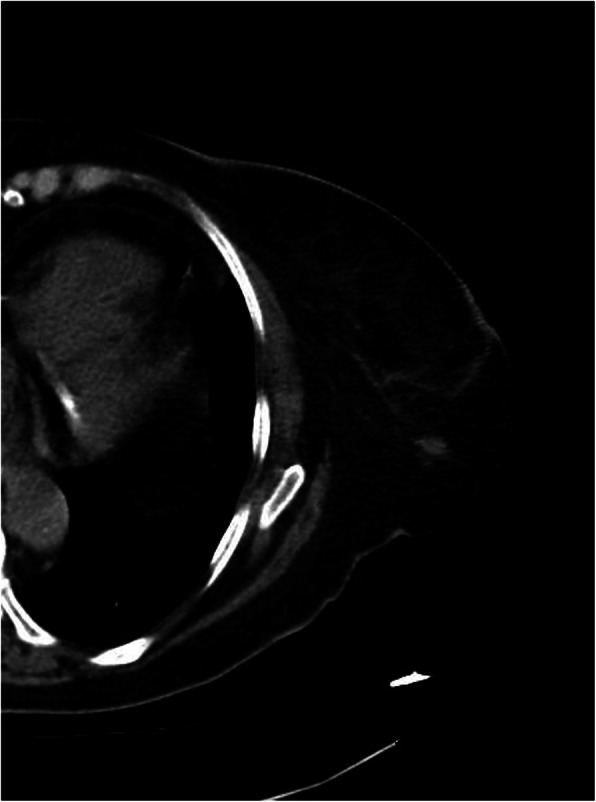
A computed tomography scan revealed nodular opacity under the left nipple and axillary adenopathy

A 72-year-old post-menopausal Caucasian woman with a positive medical history for diabetes mellitus and hypertension presented with a 3-month onset of blurred vision, diplopia, and progressive gait disturbance. A neurological examination showed severe gait and truncal ataxia preventing walking, with limbs relatively spared; pupillary responses were normal, and there was asymmetric bilateral horizontal gaze paresis (left worse than right) and horizontal nystagmus. The neurological examination was otherwise normal as well as that of her breasts and both axillary cables. She also had an excellent performance status: Eastern Cooperative Oncology Group (ECOG) 0. Routine laboratory investigations were unremarkable. A cerebrospinal fluid (CSF) examination showed mild lymphocyte pleocytosis (30 cells/mm^3^), high IgG levels (50 mg/l) with oligoclonal bands, and negative cytology and viral markers. A magnetic resonance imaging (MRI) scan revealed multiple hypointense lacunar lesions in her brainstem and near the lenticular nuclei, and diffuse alterations of the periventricular white matter (Fig. 1). Serological tests were positive for anti-Ri onconeural antibodies but negative for anti-Yo, anti-Hu, and anti-Ma. Whole-body conventional computed tomography (CT) revealed a nodular opacity of 0.8 cm under her left nipple and axillary adenopathy (Fig. 2). A carotid echo-color Doppler showed no significant alterations. A biopsy of her left breast was performed, and histological examination showed ductal carcinoma. She underwent a superoexternal quadrantectomy with left axillary dissection. The final histopathological report was consistent with the presence of multifocal grade 3 infiltrating ductal carcinoma with a high intraductal component. The tumor was estrogen receptor (ER) positive and progesterone receptor (PR) positive (ER 90%, PR 90%, Ki-67 60%) but HER2 receptor negative. The margins of the specimens were free from neoplastic infiltration. Three out of the 21 lymph nodes identified in the resected specimen were positive for ductal carcinoma. The final diagnosis showed infiltrating ductal carcinoma of the breast (T1c N1 M0, stage IIA) associated with paraneoplastic ophthalmoplegia-ataxia syndrome.

Because of lymph node positivity, we decided to treat our patient according to the doxorubicin and cyclophosphamide (AC) protocol: doxorubicin 60 mg/m^2^ administered intravenously plus cyclophosphamide 600 mg/m^2^ administered intravenously cycled every 21 days for four cycles. She received four cycles of cyclophosphamide plus doxorubicin, which were well tolerated. She was subsequently treated with docetaxel for four cycles (docetaxel 75–100 mg/m^2^ administered intravenously day 1 cycled every 21 days for four cycles) with few side effects. Moreover, due to the high expression of hormonal receptors, after chemotherapy treatment she was placed under hormonal therapy (tamoxifen 20 mg per day) without adverse effects. She also underwent adjuvant radiotherapy (40 Gy in 16 fractions). At a 6-month follow-up, there was partial clinical improvement of neurological symptoms such as ataxia and diplopia; however, some motor deterioration remained. MRI showed no signal changes in the white matter or atrophy in her brainstem, and anti-Ri antibodies were not present in the serum. Furthermore, there was no clinical or radiological evidence of neoplastic recurrence.

## Discussion and conclusion

PNSs were first described in 1968. PNSs are rare; they are estimated to occur in 0.01% of patients with cancer [3]. PNSs are generally associated with certain types of tumors, such as small cell lung cancer, thymoma, breast cancer, ovarian cancer, Hodgkin and non-Hodgkin lymphoma, testicular cancer, and neuroblastoma [7]. Breast cancer-related PNSs are very rare, in fact, Murphy *et al.* reported that only 56 cases had occurred in their institution (Mayo Clinic, USA) in the past 20 years [8]. The average age of patients was 50 years and most patients had hormone receptor (HR) positive and Her2 negative, stage II disease [8]. PNSs include paraneoplastic cerebellar degeneration (PCD), opsoclonus myoclonus syndrome (OMS), stiff person syndrome, paraneoplastic neuropathy, and paraneoplastic encephalomyelitis. These syndromes are characterized by a panel of different antibodies. These include anti-Hu, anti-Yo, anti-CV2, anti-Ri, anti-Ma2, and anti-amphiphysin. Anti-Ri antibodies are generally associated with PCD and OMS and occur in 50% and 20% of PNSs, respectively. Patients with PCD have several autoantibodies (anti-Yo, anti-Ri, anti-Tr, or anti-GluR1) that target different neuronal antigens [9]. It has been associated with small cell carcinoma of the lung, Hodgkin lymphoma, breast cancer, and gynecologic malignancies. Symptoms of PCD at presentation include subacute onset of ataxia, dysarthria, and nystagmus with ultimate progression to pancerebellar degeneration. CSF analysis shows leukocytic pleocytosis and increased IgG levels. Inflammatory changes can be detected by MRI. Paraneoplastic OMS is typically characterized by rapid, involuntary, conjugate fast eye movements (opsoclonus) and brief, involuntary twitching of muscles (myoclonus). It generally occurs with neuroblastoma but also small cell lung cancer, breast carcinoma, gastric adenocarcinoma, and renal cell carcinoma. OMS is typically associated with anti-Ri antibodies, which are directed against NOVA 1 and NOVA 2 antigens [10]. Although anti-Ri-associated PNSs occur predominantly with PCD or OMS, it has been shown that Ri PNSs are characterized by multisystem neurologic dysfunction, with a subacute or chronic progressive course. Such dysfunctions can simulate neurodegenerative or an inflammatory non-paraneoplastic condition and delay the diagnosis of the underlying tumor [11].

The diagnosis of PNSs is particularly challenging due to the variability of symptoms and the different timings of presentation. Antibodies are detected in only 70–80% of patients. However, a lack of antibodies does not exclude the presence of PNSs [12]. For this reason, a consensus of neurological experts has defined precise diagnostic criteria. These criteria are the presence of neurological symptoms, the diagnosis of cancer within 4 years of the onset of neurological symptoms, the exclusion of other neurological syndromes, and at least one of the following: inflammation with negative cytology in CSF, MRI showing a lesion in the temporal lobe, or the presence of epileptic activity in the temporal lobes as determined by electroencephalogram (EEG) [13, 14].

The immunological mechanism underlying PNSs is not well understood. It is well documented that breast cancer is highly immunogenic, and several shared tumor antigens have been identified [15]. Several studies have confirmed that loss of the p53 tumor suppressor allows unchecked cell division and permits the expression of mutated or misfolded proteins normally invisible to the immune system. The expression of aberrant antigens allows the expansion of dendritic cells, which, after scavenging cellular debris, carry new antigens through the lymphatic stream. Then, the expansion and selection of B and T lymphocyte clones occurs in the lymphoid organs, and finally autorecognition leads to autoimmune and/or paraneoplastic syndromes [16–19]. A recent study reported that the tumor microenvironment plays a decisive role in the development of humoral paraneoplastic syndromes [20]. Patients with autoreactive T lymphocytes had higher levels of interferon alpha (IFN-α) and interleukin (IL)-12 than patients who were autoantibody-negative. In particular, most probably, IFN-α supported the expansion and proliferation of T lymphocytes, which contributed to the development of PNSs [21]. In PNSs, the treatment of the underlying neoplasm is essential. Immunosuppressive treatments may help in some patients [22]. However, in some patients, the neurological symptoms progress despite the treatments. Other immunomodulatory treatments, such as intravenous immunoglobulin and plasma exchange, have achieved limited benefits in the majority of patients.

We report a rare case of anti-Ri-associated paraneoplastic ophthalmoplegia-ataxia syndrome in a 72-year-old woman. Neurological symptomatology, characterized by blurred vision, diplopia, and progressive gait disturbance, preceded the discovery of breast cancer by 3 months and simulated non-paraneoplastic inflammatory or neurodegenerative syndromes. The breast lesion was, in fact, impalpable on examination and completely asymptomatic for its small size. MRI showed nonspecific inflammatory signs characterized by hypointense lesions of the brain stem and the lenticular nuclei, and diffuse alterations of the white matter. A CSF examination showed classic inflammatory changes such as lymphocyte pleocytosis and the presence of high IgG levels. In addition, this subtype of breast cancer (ER+, PR+, HER2−) is the major one associated with PNSs. The present case illustrates that the recognition of PNSs is important, since neurological symptoms almost invariably pre-date direct symptoms of the primary tumor, and treatment at early stages may provide a better chance of a good outcome; furthermore, the presence of the anti-Ri antibody can help to identify women with opsoclonus/myoclonus and ataxia, who often suffer from breast cancer [23, 24]. We have now illustrated the occurrence of anti-Ri even in the absence of opsoclonus, thus enlarging its clinical spectrum. In this way, our findings further reinforce the belief that opsoclonus/myoclonus cannot be considered syndromic of anti-Ri antibody-associated paraneoplastic syndrome.

The diagnosis of PNSs is often late, as in this patient, but treatment at an early stage may provide a good prognosis. However, standard surgical treatment and adjuvant treatments may not cure neurological symptoms. Furthermore, this is one of several cases of anti-Ri PNSs not associated with myoclonus and it reinforces the belief that OMS is not pathognomonic of the associated anti-Ri PNSs.

## Data Availability

The datasets used and/or analyzed during the current study are available from the corresponding author on reasonable request. Data obtained for our study are publicly available under ‘Case presentation’.

## Abbreviations

AC: Doxorubicin and cyclophosphamide
PNSs: Paraneoplastic neurological syndromes
HER2: Human epidermal growth factor receptor 2
MRI: Magnetic resonance imaging
CSF: Cerebrospinal fluid
CT: Computed tomography
ECOG: Eastern Cooperative Oncology Group
EEG: Electroencephalogram
ER: Estrogen receptor
HR: Hormone receptor
IFN-α: Interferon alpha
IL: Interleukin
PCD: Paraneoplastic cerebellar degeneration
PR: Progesterone receptor
OMS: Opsoclonus myoclonus syndrome
